# The *Global Report on Adult Learning and Education (GRALE)*: Strengths, weaknesses and future directions

**DOI:** 10.1007/s11159-022-09945-x

**Published:** 2022-05-18

**Authors:** Ellen Boeren, Kjell Rubenson

**Affiliations:** 1 School of Education, University of Glasgow, Glasgow, UK; 2 Kjell Rubenson University of British Columbia, Vancouver, BC Canada

**Keywords:** *Global Report on Adult Learning and Education (GRALE)*, International Conference on Adult Education (CONFINTEA), Belém Framework for Action (BFA), adult learning and education (ALE), lifelong learning, Sustainable Development Goals (SDGs), research quality

## Abstract

One of the core outcomes of the Sixth International Conference on Adult Education (CONFINTEA VI) held in 2009 was the Belém Framework for Action (BFA). Its signatories committed to monitoring the most recent development stages of adult learning and education (ALE) worldwide on a regular basis, and to present and assess results in a global report. Coordinated by the UNESCO Institute for Lifelong Learning, surveys have been conducted and documented in four *GRALE* reports over the past decade. A fifth report is currently being prepared for CONFINTEA VII, to be held in June 2022. This article critically analyses the project of compiling a *Global Report on Adult Learning and Education (GRALE)* at roughly three-year intervals. Drawing on an evaluative framework for research quality developed by Pär Mårtensson and colleagues, the authors of this article investigate to what extent the *GRALE* approach to monitoring and reporting on ALE so far has been (1) *credible* (e.g. based on rigorous research methodologies and methods); (2) *contributory* (e.g. relevant and applicable to practice, generalisable); (3) *communicable* (e.g. accessible, understandable and readable in terms of report structure); and (4) *conforming* (e.g. with ethical standards). The purpose of this evaluation is for it to serve as a contribution to enhancing the quality of monitoring approaches in the field of ALE. This is vital for working towards future directions of ALE which are shaped by a high-quality evidence base. Ultimately, this will not only make ALE more accessible, fair, diverse and effective, but will also add to insights on how to achieve the Sustainable Development Goals in a similar way, especially since ALE indirectly but fundamentally affects the success of all 17 goals.

## Résumé

Le *Rapport mondial sur l’apprentissage et l’éducation des adultes (GRALE)* : points forts, points faibles et orientations futures – Le Cadre d’action de Belém est l’un des aboutissements principaux de la sixième Conférence internationale sur l’éducation des adultes (CONFINTEA VI) organisée en 2009. Ses signataires s’étaient engagés à procéder régulièrement au suivi des toutes dernières évolutions de l’apprentissage et de l’éducation des adultes (AEA) dans le monde entier, et à présenter et évaluer les résultats obtenus dans un rapport mondial. Des études, coordonnées par l’Institut de l’UNESCO pour l’apprentissage tout au long de la vie (UIL), ont été menées et présentées dans quatre *GRALE* ces dix dernières années. Un cinquième rapport est en préparation pour la CONFINTEA VII qui se tiendra en juin 2022. Cet article procède à une analyse critique du projet qui consiste à rédiger un *Rapport mondial sur l’apprentissage et l’éducation des adultes (GRALE)* environ tous les trois ans. S’appuyant sur un cadre d’évaluation de la qualité de la recherche développé par Pär Mårtensson et collègues, les auteurs du présent article examinent dans quelle mesure, pour procéder au suivi de l’apprentissage et de l’éducation des adultes et à la rédaction de rapports sur ce thème, l’approche du *GRALE* a été jusqu’à présent (1) *crédible* (p. ex. en s’appuyant sur des méthodes et procédés de recherche rigoureux) ; (2) *contributive* (c’est-à-dire qu’elle a joué un rôle, p. ex. en étant pertinente et praticable, généralisable) ; (3) *communicable* (p. ex. en étant accessible, compréhensible et lisible en tant que rapport) et (4) *conforme* (p. ex. aux normes éthiques). Cette évaluation a pour objet de contribuer à améliorer la qualité des approches de suivi dans le domaine de l’apprentissage et de l’éducation des adultes, ce qui est crucial pour œuvrer en vue de donner à l’AEA des orientations futures reposant sur un socle de preuves fondées. Enfin, cela rendra non seulement l’apprentissage et l’éducation des adultes plus accessibles, justes, divers et efficaces, mais cela fournira aussi des idées supplémentaires pour atteindre de la même manière les Objectifs de développement durable, du fait notamment que l’apprentissage et l’éducation des adultes influent, certes indirectement mais de façon fondamentale, sur l’atteinte des 17 objectifs.

## Introduction

This article critically analyses in a systematic way the project of presenting and assessing the most recent development stages of adult learning and education (ALE) at regular intervals in a *Global Report on Adult Learning and Education (GRALE).* Our analysis is underpinned by an evaluative research quality framework developed by Pär Mårtensson et al. (2016).

We begin by providing a historical overview of *GRALE* as a core feature of the Belém Framework for Action (BFA) (UIL 2010), which was one of the main outcomes of the Sixth International Conference on Adult Education (CONFINTEA VI) in 2009. The BFA’s signatories committed to monitoring the ongoing development of ALE,

call[ing] upon UNESCO and its structures … to coordinate, through the UNESCO Institute for Lifelong Learning in partnership with the UNESCO Institute for Statistics, a monitoring process at the global level to take stock and report periodically on progress in adult learning and education [and] to produce, on this basis, the *Global Report on Adult Learning and Education (GRALE)* at regular intervals (UIL 2010, p. 9).

In line with this mandate, the UNESCO Institute for Lifelong Learning (UIL) has conducted monitoring surveys and documented the results in four *GRALE* reports over the past decade. Already published before the BFA was drafted, the first report, *GRALE 1* (UIL 2009), served as input for CONFINTEA VI in 2009, *GRALE 2* was published in 2013 (UIL 2013), *GRALE 3* in 2016 (UIL 2016) and *GRALE 4* in 2019 (UIL 2019). UIL is currently preparing *GRALE 5* (UIL 2022), which will serve as input for CONFINTEA VII discussions in June this year. Given the end of the current CONFINTEA cycle (2009–2021), it is now time to critically evaluate the *GRALE* approach to monitoring and reporting on ALE. The purpose of undertaking this reflective task is not only to evaluate past procedures, but also to formulate recommendations on future monitoring of ALE as part of the next CONFINTEA cycle (2022–2034).

After reviewing the historical context of *GRALE* in relation to the BFA and CONFINTEA, we discuss the methodological approach of our evaluation before presenting the results of our assessment. We conclude our article with recommendations on the monitoring of ALE worldwide for future CONFINTEA cycles.

## CONFINTEA and the BFA: a brief historical overview

The series of world conferences on adult education labelled CONFINTEA (the French acronym for CONFérence INTernationale sur l’Education des Adultes), was launched in 1949, by the United Nations Educational, Scientific and Cultural Organization (UNESCO). The first conference was held 19–25 June in Helsingør (Elsinore), Denmark, in the post-war era and perceived adult education as a means to contribute to peacebuilding processes (Forrester 1998; UNESCO 1949). The dedicated conference focus on ALE has since featured at consecutive CONFINTEAs: CONFINTEA II in 1960 in Montreal, CONFINTEA III in 1972 in Tokyo, CONFINTEA IV in 1985 in Paris, CONFINTEA V in 1997 in Hamburg and CONFINTEA VI in 2009 in Belém. Upcoming CONFINTEA VII, originally scheduled to be held in Rabat in 2021, had to be postponed because of the COVID-19 pandemic. CONFINTEA is generally acknowledged to be the world’s leading forum for debating ALE policies and normative directions and is traditionally attended by a wide range of stakeholders and ALE policymakers from countries around the world.

At the 2009 CONFINTEA VI held in Belém, UNESCO Member States reiterated the fundamental role of ALE already laid out in the previous five CONFINTEA conferences, proclaiming adult education as an essential part of the right to education enshrined in the *Universal Declaration of Human Rights* (UN 1948, article 26). Attendees of the Belém conference called for a “new and urgent course of action to enable all young people and adults to exercise this right” (UIL 2010, p. 5). The direction of this agenda was developed against the backdrop of contextual factors (political and climate-related ones as well as many others besides) that had impeded the progress of ALE globally.

The outcome document of CONFINTEA VI, signed by 144 UNESCO Member States, was the *Belém Framework for Action* (BFA) (UIL 2010). The BFA points particularly to widening inequalities and rampant poverty in large parts of the world, structural shifts in the labour market resulting in growing insecurities, and adult literacy remaining a major challenge in many countries. The document also describes more subtle but complex infrastructural challenges for ALE, such as adult education being underplayed in national lifelong learning strategies; fragmentation of the field tending to make it invisible in wider social policies; underfunding compared to other educational domains; vocational ALE dominating as well as a lack of a diversity of programmes responding to the needs of different groups; and lack of professionalisation and training opportunities for adult educators having a detrimental impact on the quality of ALE processes intended to provide high-level courses and satisfy learners’ needs.

An important additional development in the interim since 2009 has been the signing of the *2015 Recommendation on Adult Learning and Education* (RALE; UNESCO and UIL 2016),^1^ which reiterates the importance of ALE in equipping adults with skills and knowledge that will help them to contribute to society both personally and professionally. RALE identifies three domains of ALE that can help adults achieve these goals: (1) *literacy and basic skills*; (2) *continuing education and vocational skills;* and (3) *liberal, popular and community education and citizenship skills* (ibid.). RALE makes a strong call to UNESCO Member States to advance the strategic directions of the BFA.

### The BFA’s five main areas of action

It could be argued that the BFA’s strategy for the realisation of the full potential of ALE represents a fundamental break with previous UNESCO reform documents on ALE and lifelong learning. In contrast to for example the reports prepared by the International Commission on the Development of Education chaired by Edgar Faure (Faure et al. 1972) and the International Commission on Education for the Twenty-first Century chaired by Jacques Delors (Delors et al. 1996; Elfert 2019), the BFA not only lays out a strong utopian normative position but combines this with a detailed instrumental administrative reform strategy. Reiterating the UNESCO mantra, the BFA expresses the conviction that ALE should equip people with

the necessary knowledge, capabilities, skills, competencies and values to exercise and advance their rights and take control of their destinies (UIL 2010, p. 6).

Further, ALE is regarded to be

an imperative for the achievement of equity and inclusion, for alleviating poverty and for building equitable, tolerant, sustainable and knowledge-based societies (ibid.).

The BFA expresses its normative position in terms of possibilities for a better type of living and represents what Jim Crowther coins as a “hopeful activity” (Crowther 2009, p. 76).

The BFA anchored its strategy for the building of a more responsive system of ALE around the world in five main areas of action: (1) *policy;* (2) *governance;* (3) *financing;* (4) *participation, inclusion and equity;* and (5) *quality*. It was through action taken in these five spheres that ALE was expected to be better able to contribute to the societal challenges and individuals’ aspirations.

#### Policy

The BFA lays out in some detail the measures UNESCO Member States committed to undertaking with regard to the areas of action. For example, it is noted that *policy* and legislative measures for ALE

need to be comprehensive, inclusive and integrated within a lifelong and life-wide learning perspective (UIL 2010, p. 7).

Countries were asked to commit to developing and implementing fully-costed policies and well-targeted plans and legislation that should be integrated into UNESCO global plans (at the time of BFA, Education for All [WEF 2000], the Millennium Development Goals [UN 2000], and the Universal Design for Learning [CAST 2008]) as well as with other national and regional plans.

#### Governance

Good *governance*, in the BFA, is seen as necessary for the implementation of adult learning and education policy in ways “which are effective, transparent, accountable and equitable” (UIL 2010, p. 7). Countries therefore set out to establish mechanisms for the involvement of public authorities at all administrative levels in ALE policies, including civil society, social partners, the private sector, learners and educational organisations. To assist non-governmental groups, the signatories resolved to undertake participation capacity-building measures. Inter-sectoral and inter-ministerial cooperation would be promoted as well as the fostering of transnational cooperation through knowledge-sharing cooperation.

#### Financing

*Financially*, UNESCO Member States agreed to

accelerat[e] progress towards achieving the CONFINTEA V recommendation to seek investment of at least 6% of GNP [gross national product] in education (UIL 2010, p. 7)

and to expand this investment across all departments to meet the objectives of an integrated ALE strategy and to strengthen transnational funding. ALE is seen as a

valuable investment which brings social benefits by creating more democratic, peaceful, inclusive, productive, healthy and sustainable societies (UIL 2010, p. 7).

Prioritised “investment in learning for women, rural populations and people with disabilities” was called for (ibid.), and cooperation with international partners was recommended to fill those gaps preventing the achievements of all international development goals.

#### Quality

*Quality* in learning and education is seen by the BFA as a

holistic multidimensional concept and practice that demands constant attention and continuous development. Fostering a culture of quality in adult learning requires relevant content and modes of delivery, learner-centred needs assessment, the acquisition of multiple competences and knowledge, the professionalisation of educators, the enrichment of learning environments and the empowerment of individuals and communities (UIL 2010, pp. 8–9).

To foster quality in ALE, UNESCO Member States committed to developing quality criteria for curricula, learning materials and teaching methods and to act on outcomes and impact measures. The diversity and plurality of providers was to be recognised, training and work towards professionalisation of adult educators were to be improved, quality indicators were to be developed, interdisciplinary research in ALE was to be strengthened and complemented by a knowledge management system for the collection, analysis and dissemination of data and good practice.

#### Participation, inclusion and equity

The domain of *participation, inclusion and equity* differs from the other four in that it can be seen more as the aspired outcome and overall goal of the other four areas of action. It therefore mostly repeats actions identified under the other four areas, tailored towards increasing participation in ALE around the globe. Of the specific activities not covered under the other areas of action it resolves to provide well-designed and targeted guidance and information activities. Its focus on inclusion and equity is important and stressed by underlining that there can be

no exclusion arising from age, gender, ethnicity, migrant status, language, religion, disability, rurality, sexual identity or orientation, poverty, displacement or imprisonment (UIL 2010, p. 8).

### The emergence of the monitoring project

In accordance with the evidence-based policy movement that had come to dominate the public policy landscape in the first decade of the 21st century, monitoring was central to the BFA agenda. UNESCO Member States acknowledged the need for valid and reliable data to inform policymaking in ALE and to facilitate policy transfer, and they committed to implementing appropriate monitoring mechanisms. Thus, countries promised to develop appropriate measurement instruments, regularly collect and analyse data on participation and progress in ALE and to prepare a triennial status report to be submitted to UNESCO. These data form the basis of the *Global Report on Adult Learning and Education (GRALE).* Not only does *GRALE* focus on national data, it largely presents these data in their regional context and also includes presentation of data by country income level groupings (a classification used by the World Bank).^2^ In addition to these geographical and economic dimensions, there is the longitudinal perspective. The report focuses on the need to track the evolution of ALE progress over time. This strategy can be seen as a reaction to criticism of the ALE community for repeatedly putting forward a utopian agenda mainly devoid of attention to the practicalities of its implementation or accompanied by a realistic policy agenda. By focusing on “what works” and hard evidence, attempts could now be made to avoid an agenda driven almost entirely by ideology or values.

When critically analysing the *GRALE* monitoring and reporting project it should be noted that CONFINTEA conferences are “level 2” conferences in the United Nations (UN) hierarchical scheme. This means that their conclusions are recommendations and that these are therefore not binding on governments. They are norms which are not subject to ratification, but which UNESCO Member States are invited, indeed encouraged to apply if they wish to. Both the Belém conference (CONFINTEA VI) in 2009 and the mid-term review meeting held in Suwon in 2017 were attended by relatively few ministers, especially from member countries of the Organisation for Economic Co-operation and Development (OECD). This observation raises questions in terms of how much pressure there has been in some of the UNESCO Member States to realise the Belém Framework for Action. Similarly, the original brief to UIL on the monitoring of the implementation of the BFA could have been strengthened by including a request for evidence of the impact the monitoring process was having on the ground. As we argue below, one of our core recommendations for the next CONFINTEA cycle is a stronger focus on impact or the need to carry out an in-depth impact evaluation.

## Methodological approach

This section provides a brief overview of the methodological approach this article is based on, with our analysis being underpinned by an evaluative framework developed by Pär Mårtensson et al. (2016). Based on this framework, we investigate to what extent the *GRALE* approach to monitoring and reporting on ALE has been (1) credible, (2) contributory, (3) communicable and (4) conforming, which are categories that will be explained.

**Fig. 1:**
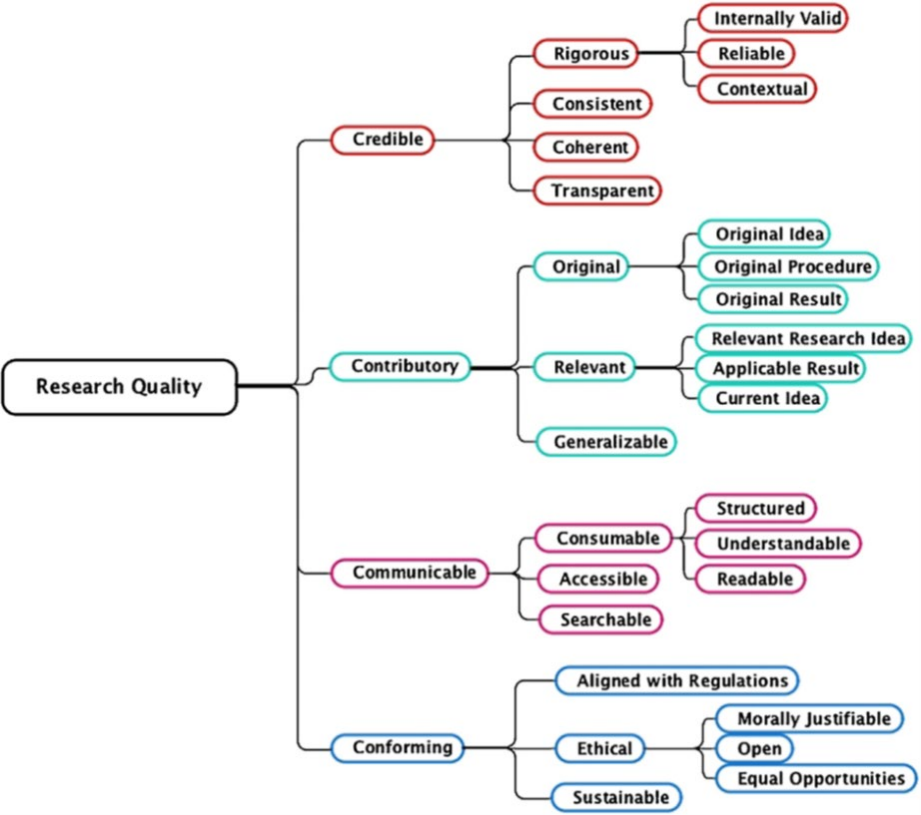
Concept hierarchy of research quality (Mårtensson et al. 2016)

The framework developed by Mårtensson et al. (2016) resulted from a group of senior academics at a Swedish university who collaborated on “research quality concept modelling”. Members of this working group met eight times and all of them had extensive research experience from a wide variety of universities in Europe, America and Australasia. They worked in a wide range of academic fields such as medicine and health sciences, exact sciences and social sciences, arts and humanities. The aim of the working group was to achieve a common understanding of “research quality” applicable across the different disciplines. This work resulted in a map of 32 concepts, clustered into four areas (see Fig. 1). These formed the basis of our evaluation criteria in this article. As recommended by Mårtensson et al. (2016), one of the uses of this framework is that it is particularly suited to evaluating existing research against these criteria. As shown in Fig. 1, Märtensson et al. broke down the first category, “credible”, into four concepts, and each of the other three categories (“contributory”, “communicable” and “conforming”) into three concepts respectively.

Using the research quality framework developed by Mårtensson et al. (2016), we undertook our evaluation of the *GRALE* monitoring and reporting project based on our experiences as editors and authors of one or more *GRALE* reports and backed up by evidence from *GRALE* reports, the *GRALE* sections on the UIL website and further available material about the reports. As such, our approach to the evaluation might be best described as a document analysis.

## Evaluation of the *GRALE* monitoring and reporting project

### Credibility

Credibility strongly relates to aspects of research rigour and includes elements of validity and reliability. The evaluation framework we use in this article also refers to consistency, coherence and transparency. *GRALE*’s primary data collection is done through a standardised questionnaire (see UIL 2021a, where links are provided to each successive questionnaire) which is sent out to UNESCO Member States and affiliated countries by UIL. This self-reporting survey provides a global snapshot of the most recent development stages of ALE through monitoring the BFA’s five areas of action.

While the survey uses a standardised instrument, several weaknesses in relation to validity and reliability can be identified. A major issue with the *GRALE* survey is that it only requires one return per country. UIL has limited control in relation to the quality of the data and it is unclear whether countries have their own quality control mechanisms in place such as moderation of responses among the multiple respondents, often different types of stakeholders, who have delivered input to the responses. To generate insight into who has been involved in completing the survey, some data have therefore been collected in recent years by adding a multiple-choice question with checkbox categories. For example in the questionnaire collecting data for *GRALE 4*, options available included civil servants working for government departments such as Education, Social Affairs, Health or Labour, colleagues active in education-related NGOs, workers at research institutes or universities or private sector workers.

To further increase the quality of country reports for *GRALE 5*, regional webinars were held in the preparation phase, offering guidance to UNESCO Member States’ designated contact persons (the UNESCO term is “focal points”) on how to complete their surveys. Nevertheless, the approach of one response per country runs the risk of painting an inaccurate picture of what is going on and ideally needs to be supplemented by in-depth documentary analyses for countries participating in the study, for example through a more thorough analysis of available secondary data or a more robust inspection of national policy reports.

The questionnaire consists of standardised closed-ended as well as a limited number of open questions. While the former are quicker to extract and process for quantitative inclusion in *GRALE*, the latter give respondents the opportunity to comment in their own words, in some cases providing valuable qualitative data. The final survey instrument is the result of extensive discussions between the *GRALE* team at UIL and external consultants who work as active researchers in the field of ALE. The aim of these discussions is to ensure the validity of the instrument to a certain extent, although it remains unclear whether respondents in different countries truly interpret survey questions in the same way. While the aim of adapting and refining the questionnaires between the different monitoring phases is to improve data, it runs the risk of limiting the comparability of data between *GRALE* reports, which in turn might limit coherence of *GRALE* reporting within the CONFINTEA cycle. Among the adjustments made from *GRALE 4* (UIL 2019) onwards, for example, were questions and response options explicitly introducing the three categories of RALE: (1) *literacy and basic education;* (2) *continuing and vocational education and training;* and (3) *citizenship education*. Nevertheless, attempts have been made to draw comparisons between the different *GRALE* cycles and to report trends over time.

Including this longitudinal reporting perspective as input for CONFINTEA VII will also be a major aim of *GRALE 5* (UIL 2022). Questionnaire items for this latest *GRALE* report contain many ALE-related terms such as “literacy”, “basic skills”, “non-formal learning” and “informal learning”, among others. After extensive discussion by the *GRALE* team responsible for questionnaire design, definitions for each of these concepts were included in the questionnaire to provide clarity to the respondent of what is meant. This procedure is especially important given the global scope of *GRALE* with countries from all world regions taking part in this monitoring exercise. Concepts can be highly contextual and therefore need some standardisation to avoid comparing apples with oranges.

The *GRALE* survey tends to be transparent in that it is upfront about measuring progress towards the five domains outlined in the BFA:(1) *policy;* (2) *governance;* (3) *financing;* (4) *participation, inclusion and equity;* and (5) *quality.* The question format tends to be coherent between the different domains of the BFA, and similar structures have been used consistently between the different *GRALE* phases. Overall, the main weakness in relation to credibility lies in *GRALE*’s overreliance on one response per country, which is not supported by any built-in mechanism for quality control.

### Contributoriness

The evaluation framework we use in this article defines the “contributory” part of research quality according to three points: whether the research is (1) *original*, (2) *relevant* and (3) *generalisable*. *GRALE* does indeed make an *original* contribution to the field of ALE, since it is the leading global monitoring operation in the area. While organisations like OECD and the European Commission also monitor elements of ALE – for example participation rates of adults in education and training activities – *GRALE* does a unique job in monitoring the areas of the BFA and does so across a wider range of countries. While the procedure it uses through a country-level survey is not very innovative, it does lead to a set of data that no other organisation has generated.

The *relevance* of *GRALE* has been made explicit over the years through reflections on earlier ALE documents produced by UIL, stating the importance of ALE as part of personal development, creating a sense of belonging to society, improving levels of literacy and working towards qualifications. At the time of CONFINTEA VI, reflections were related to the United Nations Education for All agenda (2000–2015) with its eight Millennium Development Goals (UN 2000), none of which explicitly addressed adult education. Meanwhile, the discourse has been replaced by references to the 2030 Agenda (2015–2030) with its 17 Sustainable Development Goals (UN 2015).

To what extent *GRALE* results have direct applicability to new developments in ALE is difficult to judge, since we have been unable to find any rigorous impact evaluations in the public domain, for example through searching for them using university library catalogues or search engines. If any such impact evaluations were to be made, we would expect them to find that while *GRALE* does contribute to debates on ALE in the Global South, major policy influences in the Global North are more likely driven by metrics and discourses initiated by the European Commission and survey programmes like the OECD’s Programme for the International Assessment of Adult Competencies (PIAAC) (Ball 2017). UIL has in fact undertaken specific efforts to enable *GRALE* to contribute to change in African countries and has published a separate report on ALE trends on the African continent (UIL 2020). This has led to key messages to African ALE policymakers and stakeholders conveying that participation remains uneven with large social inequalities between different socio-economic and socio-demographic groups, that the lack of reliable ALE data hinders evidence-based changes, that ALE remains an underfunded domain and that governance of ALE continues to be poor. Progress tends to be slow, and more patience is needed to see improvements made within the *GRALE 4* and *5* phases. Nevertheless, *GRALE* – as well as monitoring achievements towards reaching SDG targets – could be seen as a sensible way of maintaining peer pressure on countries.

In terms of whether its research is *generalisable*, one of the weaknesses of *GRALE* under the “contributory” criterion of research quality lies in its practice of reporting by world regions and country income level groups. Some of these groups are broad and lump together countries that have significantly different ALE characteristics. This is for example the case for the “North America and Western Europe” group, which includes Nordic ALE systems as well as Mediterranean ones. Critical questions need to be asked about how far results of these groupings can be generalised to the situations and contexts of countries that did not participate in the *GRALE* survey. Based on studies of political economies of adult learning systems (see Boeren 2016; Desjardins 2017), a reluctance to generalise needs to be introduced.

Similarly, a more critical approach needs to be adapted to dangers of overclaiming if certain regional groupings contain a high proportion of missing values. There is a tendency in the *GRALE* monitoring approach to somewhat uncritically accept and overstate its findings. As an example, we can look at how the relationship between funding and participation is being presented in *GRALE 4* (UIL 2019). According to the results (ibid., Table 1.15, p. 75) 72 per cent of countries in sub-Saharan Africa reported an increase in their overall ALE participation rate since 2015, higher than in any other region. However, of the 32 countries in this region which had responded to the question, only 22 had actual data on participation. Further, several of the sub-Saharan countries had not been able to participate in the survey at all. Thus, the impressive figure of 72 per cent reporting an increase is based on actual data from slightly less than half of the countries in the region. The way the results are being presented puts the burden on an observant reader to critically evaluate what the data really stand for. A more sceptical attitude towards the results is something all of the *GRALE* reports would have benefited from. This uncritical approach is further accentuated by a lack of cross-tabulation of the various findings that could have helped with the interpretation.

Finally, the descriptive nature of reporting on the five domains of the BFA limits the understanding on how they interact with each other. For example, it would be interesting to see what the intersection is between UNESCO Member States’ approaches to policy and governance, or financing and participation. While data are available to engage with these questions, these types of analyses have not been undertaken as part of the *GRALE* monitoring and reporting project.

### Communicability

While *GRALE* is a written report, it has been supplemented throughout the years by seminars and events. Printed versions of the report are available although it can also be consulted online in an open access format. The working language of *GRALE* is English, and the report is written in an accessible style, also bearing in mind that many international readers’ first language is not English. Subsequent translations of the English version also make the reports available in a range of other languages. For example, *GRALE 4* can be downloaded from the UIL website/UNESCO’s “unesdoc” digital library in Arabic, French, German, Korean, Russian, Portuguese, Russian and Spanish. Given its online availability, it is clear that *GRALE* is accessible to stakeholders and policymakers in all corners of the world who typically will have access to an internet connection as part of their work.

While the report is thus openly accessible, it is important to reflect on how far it is consumable. Recent *GRALE* reports have followed a similar structure, starting with an Introduction, typically followed by Part 1 containing a reflection on survey results as part of the monitoring of the BFA domains. Part 2 then zooms in on a thematic focus before the report culminates in a Conclusion. Generally speaking, this is a logical structure. However, whether the information presented in the report is in fact digestible needs to be evaluated with its end users. As researchers working in the field, we could argue that some of the statistics presented in the report are too condensed as they are presented by world regions and country income level groups instead of being displayed by country. This makes it difficult for national policymakers and stakeholders to compare their own country’s performance against that of others, or against their performance in past reports.

In addition to providing online PDF files of the successive editions of the printed report, UIL also maintains a dedicated *GRALE* webpage where it hosts Excel and SPSS datasets on countries’ responses to the survey (UIL 2021a). It is unclear to what extent end users are familiarising themselves with these data and whether they are using them to build a customised evidence base for their own ALE work. Country responses to the *GRALE 1, 2* and *3* surveys are also available through UIL’s website (UIL 2021b), readily accessible for researchers or stakeholders to engage in secondary analyses.

Although executive summaries have been produced for each *GRALE* report and the Introduction and Conclusion sections set out core take-away messages, the report itself is lengthy, ranging from 156 pages (*GRALE 1*) to 195 pages (*GRALE 4*). While the *GRALE* report is largely quantitative in nature, presenting results of the survey, several findings are highlighted by boxed case studies throughout the text (e.g. “Box 2.13 AdulTICo Program [Colombia]”, UIL 2019, p. 143). The items thus featured can, generally speaking, be interpreted as best practices.

Over the years, UIL has also organised seminars in relation to *GRALE*. While the ultimate usefulness of these seminars needs to be evaluated with their attendees, it is positive to see engagement with stakeholders that goes beyond the level of a written report. During the pandemic, webinars were organised, for example to support national stakeholders in completing their *GRALE 5* surveys. Similar events were organised to disseminate *GRALE* findings and to engage in a debate about how to make progress towards the realisation of the full potential of ALE as envisaged in the BFA. One major event in recent years was the CONFINTEA mid-term review held in Suwon, Korea, in 2017 (UIL 2018).

While *GRALE* has thus gained some attention at the policy level, albeit less so from OECD member countries, it has not really “arrived” in academic circles. Our analyses of Scopus and EBSCOhost searches revealed that fewer than ten academic articles have been published on *GRALE*. For example, an Academic Search Premier analysis on EBSCOhost led us to discover 7 articles on *GRALE*, but over 10,000 articles on the OECD’s Programme on International Student Assessment (PISA). To address this dearth of *GRALE*-related academic literature, one way forward might be to initiate a global research programme on *GRALE* involving academics, stakeholders at the level of policy and practice and UIL itself.

### Conformity

The “conforming” notion of research quality in the framework refers to the need for research projects to be ethical and to comply with regulations. UIL sends the survey questionnaire to designated national contact persons (“focal points”) of all 193 UNESCO Member States and 11 Associate Members and gives each of them an equal chance to participate in the survey. The survey questionnaires are available in all six official UN languages (Arabic, Chinese, English, French, Russian and Spanish). Countries are free to return their questions in the language of their choice. All Member States are encouraged to participate, but participation remains voluntary, which is reflected in the non-response from several countries around the world. For example, for *GRALE* 4, the United Kingdom did not return their questionnaire. Nevertheless, a response rate of 80 per cent was achieved.

Scrutinising the report and the available documentation on the website – for example the *GRALE 3* questionnaire (UIL 2015) – it needs to be noted that no explicit references are made to standard ethical procedures, such as informed consent sheets. Survey responses are made publicly available on UIL’s website and there is thus no guarantee of anonymity. UNESCO Member States are informed that data are being sourced to be presented in the *GRALE* report, and UIL’s approach to data collection can therefore be justified as being open and upfront, but ethical procedures could be communicated in a more transparent way. Work undertaken towards delivering *GRALE* reports falls under UIL’s legal status as a Category 1 UNESCO Institute (a fully-fledged component of the organisation), for which a cooperation agreement was signed between UNESCO, the German government and the City of Hamburg in 2006.

## Discussion of evaluation

In this section, we summarise our considerations to provide a short overall assessment of *GRALE*, blending together insights from our evaluation using the four domains of the research quality framework designed by Mårtensson et al. (2016). In short, we judge the quality of *GRALE* based on the approaches used to collect data as preparation for the reports as well as the ways in which *GRALE* is being presented to the wider world.

First of all, we think it is a fair assessment to raise some concerns about the credibility of the *GRALE* approach to monitoring ALE. Its overreliance on self-reporting among countries runs the risk of presenting misinformation and there are no quality control mechanisms in place to check the validity and reliability of what survey responses claim. Issues around credibility also relate to the ways in which ALE concepts are being measured, as discussed in the previous section.

Second, it is unclear how far *GRALE* has been truly contributory to advancing a realisation of the full potential of ALE as envisaged in the BFA. While the idea of a triennial global report is relevant, it is difficult to come to a conclusion about the extent to which it has really enabled change in society.

Third, it needs to be acknowledged that UIL has undertaken efforts to make the *GRALE* reports visible to stakeholders around the world through summary versions of the report, through translations into a number of languages, complemented by seminars held in different world regions, and online workshops during the COVID-19 pandemic. Yet the presentation of information by larger world regions and country income level groups diminishes its utility for individual countries.

Fourth, while we were unable to find any major issues with UIL’s “conforming” approaches, from an academic research perspective, ethical standards could potentially be made more explicit to stakeholders completing the survey.

Overall, while *GRALE* is an important monitoring and reporting project, there is considerable room for improvement if it wants to become an instrument for evidence-based policymaking. Based on these insights, we formulate a number of recommendations for the future of *GRALE*.

## Recommendations for the future of ALE monitoring

We are now at the end of a CONFINTEA cycle, with CONFINTEA VII now (re)scheduled to be held in Marrakech in June 2022. As mentioned above, the conference had to be postponed given the COVID pandemic. Just over 12 years ago, the signing of the BFA was a core outcome of CONFINTEA VI in Belém. Taking on board the BFA’s five areas of action, UIL has continued its work on fulfilling its mandate to monitor the most recent development stages of ALE on a regular basis and to present the results of this monitoring in global reports. This work is about to mark its next stage by presenting *GRALE 5* to UNESCO Member States during the upcoming world confeence. At the moment, it is unclear whether the outcome of CONFINTEA VII will encourage continuation of the *GRALE* approach or other monitoring practices, although it seems likely that some sort of continuation will be proposed. As scholars in the field of ALE, and as past contributors to *GRALE*, we would welcome this suggestion. At the same time, the purpose of this article is to make four recommendations to help improve the monitoring process.

### The need for a strong problem statement

First of all, monitoring practices should ideally start from a situation with a strong problem statement. What are the major societal challenges countries are dealing with? Why are these problems present and what is the role of ALE in supporting sustainable change in society? Starting from answers to these questions could help to flesh out and deepen links to the Sustainable Development Goals going forward. This would give stakeholders more profound insight into the “so what?” value of the report. This could then act as a stronger starting point for asking critical questions about the most recent development stages of ALE. Why is ALE important and why do some UNESCO Member States not succeed in having strong ALE processes in place? What exactly is it in relation to policy, governance, financing, participation and quality that is not working well in different countries? Ultimately, it will be important to ask the question of how far ALE can help in dealing with major societal challenges, and to better understand the need to build partnerships with providers of other types of social interventions that are not typically classified as ALE.

### The need to factor in correlations among the five BFA domains

Second, one of *GRALE*’s weaknesses is the segregated presentation of the five BFA domains. For example, the reader gets to see an overview of survey results in relation to policy and to finance, but there is no presentation of how these domains correlate with each other. It is highly likely that some sort of logic links them but, to date, an in-depth analysis scrutinising the evidence on this is lacking from the knowledge base.

### The need to initiate a thorough impact evaluation

Third, we recommended undertaking a thorough impact evaluation to investigate what *GRALE* has achieved on the ground. Has it effected any change in relation to the most recent development stages of ALE in individual countries? Has *GRALE* enabled changes in ALE that have helped to make progress towards reaching the Sustainable Development Goals? If societal change occurred, to what extent can it be attributed to *GRALE*? What other interventions might have led to change? These questions are currently unanswered. A large-scale research project might provide answers to these questions, although smaller investigations such as single-country studies could be undertaken in this area too.

### The need to become more evidence-based and avoid the image of an advocacy project

Finally, fourth, the methodologies and methods, including measurements of ALE as part of the survey, need to be scrutinised, to avoid the risk of *GRALE* solely being an advocacy project that aims to boost and encourage the profile of ALE without being underpinned by a robust impact evaluation. How far do research quality issues on “credibility” stand in the way of achieving truly evidence-based recommendations? To what extent does *GRALE* represent the reality of ALE on the ground? What work can be undertaken to further unpack the true meaning of RALE’s three ALE categories, namely (1) *literacy and basic skills;* (2) *continuing education and vocational skills;* and (3) *liberal, popular and community education and citizenship skills* – also in terms of how they interrelate and how they can be measured? It is only through increased levels of rigour, validity and reliability in *GRALE*, and growing the research base on ALE, that stakeholders will be able to get a better sense of the direction of travel.

## Footnotes

1 RALE replaced an earlier *Recommendation on the Development of Adult Education* (UNESCO 1976), an update of which had been proposed during both CONFINTEA V in 1997 and CONFINTEA VI in 2009.

2 “The World Bank assigns the world’s economies to four income groups – low, lower-middle, upper-middle, and high-income countries. The classifications are updated each year on July 1 and are based on GNI per capita in current USD … of the previous year” (Hamadeh et al. 2021).
